# Supplementary material for: Functional synergy of a human-specific and an ape-specific metabolic regulator in human neocortex development
Source: Nat Commun. 2024 Apr 24;15:3468. doi: 10.1038/s41467-024-47437-8 (PMC11043075; doi:10.1038/s41467-024-47437-8)
Supplement: Supplementary file 1 — Supplementary Information [file 41467_2024_47437_MOESM1_ESM.pdf]

Supplementary Information

Functional synergy of a human-specific and an ape-specific metabolic regulator in human neocortex development

Lei Xing, Vasiliki Gkini, Anni I Nieminen, Hui-Chao Zhou, Matilde Aquilino, Ronald Naumann, Katrin Reppe, Kohichi Tanaka, Peter Carmeliet, Oskari Heikinheimo, Svante Pääbo, Wieland B. Huttner, Takashi Namba

Supplementary information contains:

Supplementary figures and legends (S1 – S9)

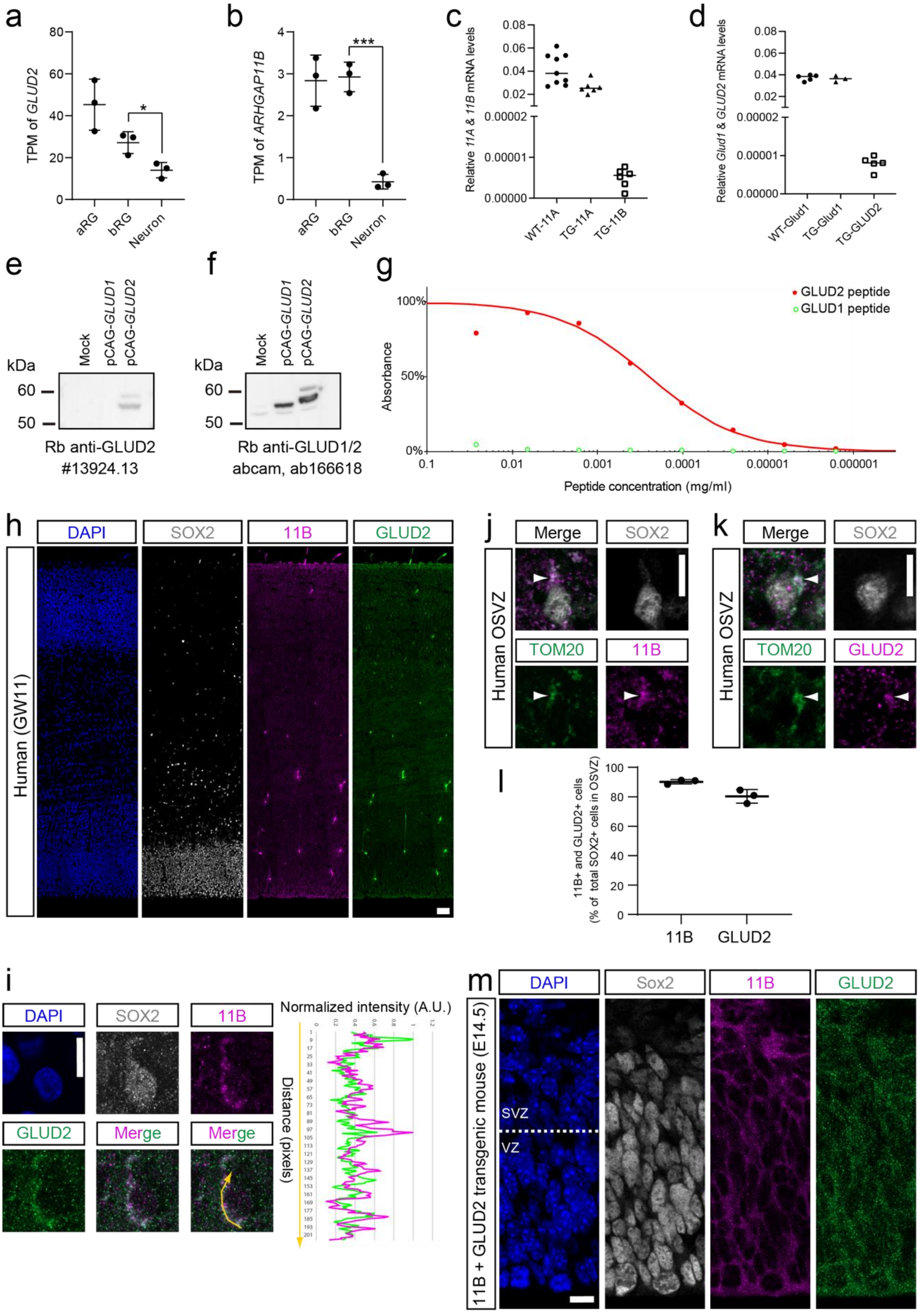

Supplemental figure 1. GLUD2 and ARHGAP11B expression in fetal human and

ARHGAP11B+GLUD2 double-transgenic embryonic mouse neocortex.

a, **b**, *GLUD2* (a) and *ARHGAP11B* (b) expression in aRG, bRG and neurons in the fetal human neocortex. Re-analysis of the RNA sequencing data (GSE66217[52]). Values are indicated as transcript per million (TPM).

c, qPCR analysis of *Arhgap11a* expression in the neocortex of WT (WT-11A) and *ARHGAP11B*-transgenic (TG-11A) mouse embryos and of *ARHGAP11B* expression in the neocortex of *ARHGAP11B*-transgenic mouse embryos (TG-11B), all at E14.5.

d, qPCR analysis of *Glud1* expression in the neocortex of WT (WT-Glud1) and *GLUD2*-transgenic (TG-Glud1) mouse embryos and of *GLUD2* expression in the neocortex of *GLUD2*-transgenic mouse embryos (TG-GLUD2), all at E14.5.

e, f, Validation of the newly generated anti-GLUD2 antibody by immunoblotting. COS7 cell lysates after transfection of either pCAG-empty (Mock), pCAG-*GLUD1* or pCAG-*GLUD2* were subjected to immunoblotting using either the newly generated rabbit (Rb) anti-GLUD2 antibody 13924.13 (**e**) or, for comparison, the commercially available rabbit anti-GLUD1/2 antibody ab166618 (**f**).

g, Validation of the newly generated rabbit anti-GLUD2 antibody 13924.13 by ELISA using either a GLUD2 (red dots) or GLUD1 (green dots) peptide (for details, see Methods) as antigens at the indicated concentrations.

h, **i**, Immunofluorescence of fetal (GW11) human neocortex for SOX2 (white), ARHGAP11B (11B, magenta) and GLUD2 (green), with DAPI staining (blue). A SOX2+ARHGAP11B+GLUD2+ cell in the OSVZ at higher magnification is shown in (**i**). The right-most panel in (**i**) shows the normalized signal intensity (for details, see Methods) of ARHGAP11B (magenta) and GLUD2 (green) immunofluorescence along the yellow arrow as indicated.

j, **k**, Immunofluorescence of fetal (GW15) human OSVZ for SOX2 (white), TOM20 (green), and ARHGAP11B (11B, magenta in **j**) or GLUD2 (magenta in **k**). Arrowheads indicate the proximal part of a basal process.

l, Quantification of the percentage of SOX2+ cells in the fetal (GW15-17) human OSVZ that express ARHGAP11B (11B) and GLUD2, using immunostained cryosections as obtained in (**j**, **k**).

m, Immunofluorescence of embryonic (E14.5) mouse neocortex for Sox2 (white), ARHGAP11B (11B, magenta) and GLUD2 (green), with DAPI staining (blue). ARHGAP11B and GLUD2 immunofluorescence signals are observed in the Sox2+ cells located in the VZ and SVZ. The dashed lines indicate the border between the VZ and SVZ.

Error bars, SD; Scale bars: 100 µm in **h**; 10 µm in **I**, **j**, **k**, **m**.

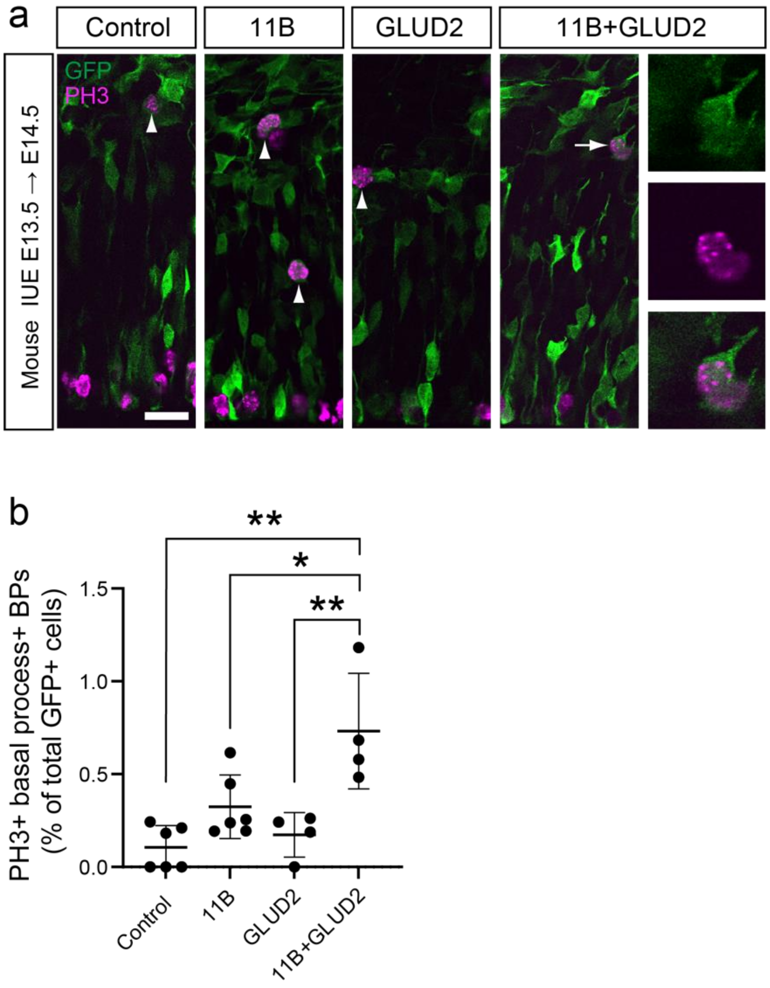

Supplemental figure 2. Greater abundance of mitotic bRG in *ARHGAP11B* plus *GLUD2* cDNA-transfected than *ARHGAP11B* cDNA-transfected wildtype embryonic mouse neocortex.

Wildtype embryonic mouse neocortex at E13.5 was electroporated with a plasmid encoding GFP, together with either an empty vector (Control), an ARHGAP11B-expressing plasmid (11B), a GLUD2-expressing plasmid (GLUD2), or ARHGAP11B- plus GLUD2-expressing plasmids (11B+GLUD2).

a, Immunofluorescence of Control, 11B, GLUD2 and 11B+GLUD2 embryonic mouse neocortex for GFP (green) and phosphorylated histone H3 (PH3, magenta). Arrow, GFP+PH3+ cell with a basal process (i.e., a bRG); the panels on the right show this cell at higher magnification (top, GFP; middle, PH3, bottom, merge). Arrowheads: GFP+PH3+ cells without a basal process (i.e., bIPs). Scale bar, 20 µm.

b, Quantification of the percentage of abventricular GFP+ cells that are PH3+ cells with a basal process (bRG) in control, 11B, GLUD2 and 11B+GLUD2 embryonic mouse neocortex, using immunostained cryosections as obtained in (**a**). Error bars, SD; *p<0.05; **p<0.01.

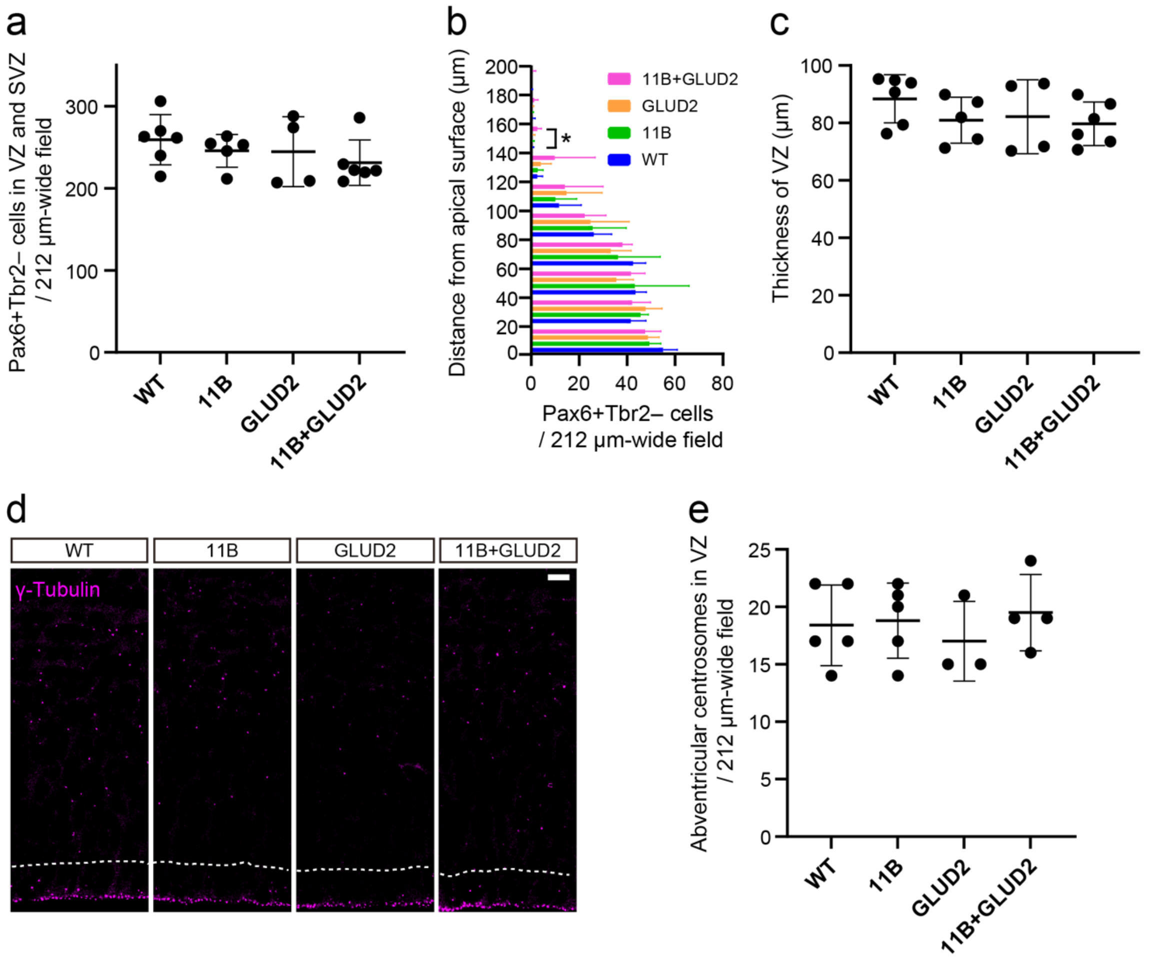

Supplemental figure 3. NPC abundance and possible aRG delamination, and thickness of the VZ.

a, Quantification of Pax6+Tbr2– cells in the VZ and SVZ of E14.5 wildtype (WT), *ARHGAP11B*-transgenic (11B), *GLUD2*-transgenic (GLUD2) and *ARHGAP11B+GLUD2* double-transgenic (11B+GLUD2) mouse neocortex, using immunostained cryosections obtained as in Figure 1f.

b, Quantification of the distribution of Pax6+Tbr2– cells along a 200 µm radial axis (divided into 10 bins) of E14.5 wildtype (WT, blue), *ARHGAP11B*-transgenic (11B, green), *GLUD2*-transgenic (GLUD2, orange) and *ARHGAP11B+GLUD2* double-transgenic (11B+GLUD2, magenta) mouse neocortex, using immunostained cryosections obtained as in Figure 1f.

c, Quantification of the thickness of the VZ of E14.5 wildtype (WT), *ARHGAP11B*-transgenic (11B), *GLUD2*-transgenic (GLUD2) and *ARHGAP11B+GLUD2* double-transgenic (11B+GLUD2) mouse neocortex.

d, Immunofluorescence of E14.5 wildtype (WT), *ARHGAP11B*-transgenic (11B), *GLUD2*-transgenic (GLUD2) and *ARHGAP11B+GLUD2* double-transgenic (11B+GLUD2) mouse neocortex for γ-tubulin (magenta). Dashed lines indicate the distance of 15 µm from the apical surface.

e, Quantification of abventricular γ-tubulin+ centrosomes in the VZ; an abventricular location was defined when centrosomes were located at least 15 µm basal to the apical surface

Error bars, SD; * $p<0.05$; Scale bar: 10 µm in **d**.

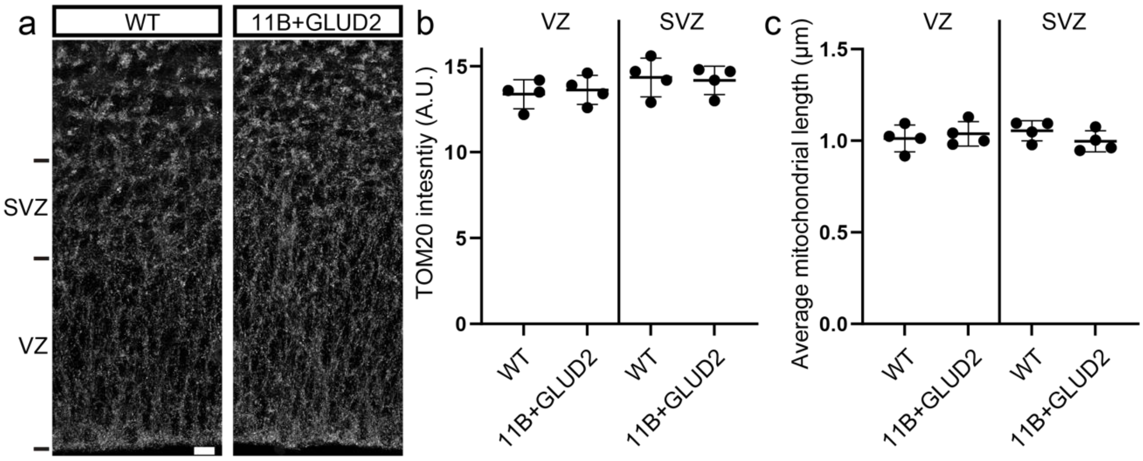

Supplemental figure 4. Mitochondria abundance and morphology are not changed in ARHGAP11B+GLUD2 neocortex.

a, Immunofluorescence of E14.5 wildtype (WT) and *ARHGAP11B+GLUD2* double-transgenic (11B+GLUD2) mouse neocortex for the mitochondrial marker protein TOM20.

b, Quantification of TOM20 immunofluorescence signal intensity in the VZ (left) and SVZ (right).

c, Quantification of TOM20+ mitochondrial length in the VZ (left) and SVZ (right).

Error bars, SD; Scale bar: 10 µm in **a**.

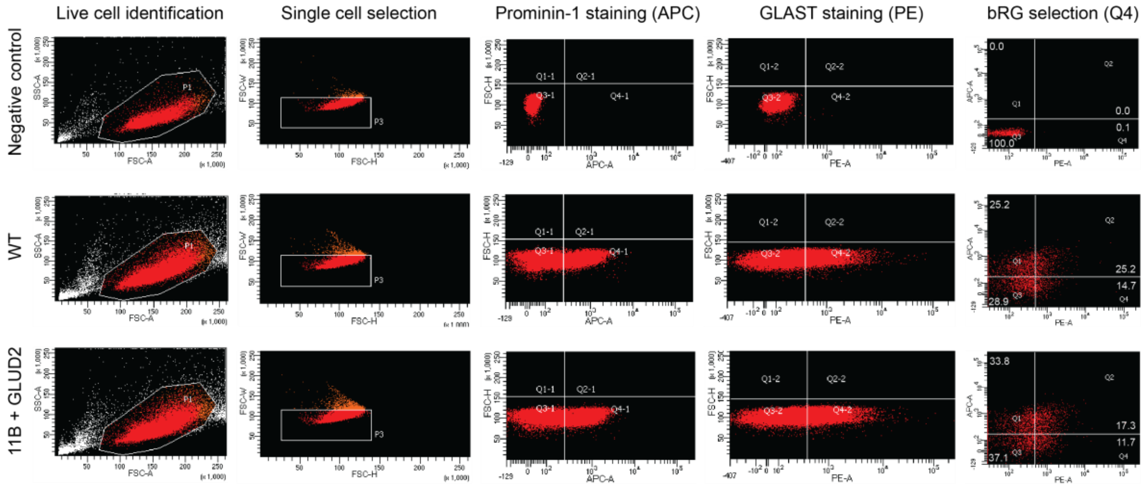

Supplemental figure 5. Fluorescence-activated cell sorting for isolating bRG from the developing mouse neocortex. Stringent gating has been used to guarantee the purity and quality of the isolated bRG. Note that the gatings in the Prominin-1 staining and GLAST staining panels were set not right next to, but far from, the unstained cell population in the negative control, and the gatings in the single cell selection panels were set to eliminate any potential duplet cells. The values in the right-most panels indicate the proportion of the P3 cells in each quadrant (%).

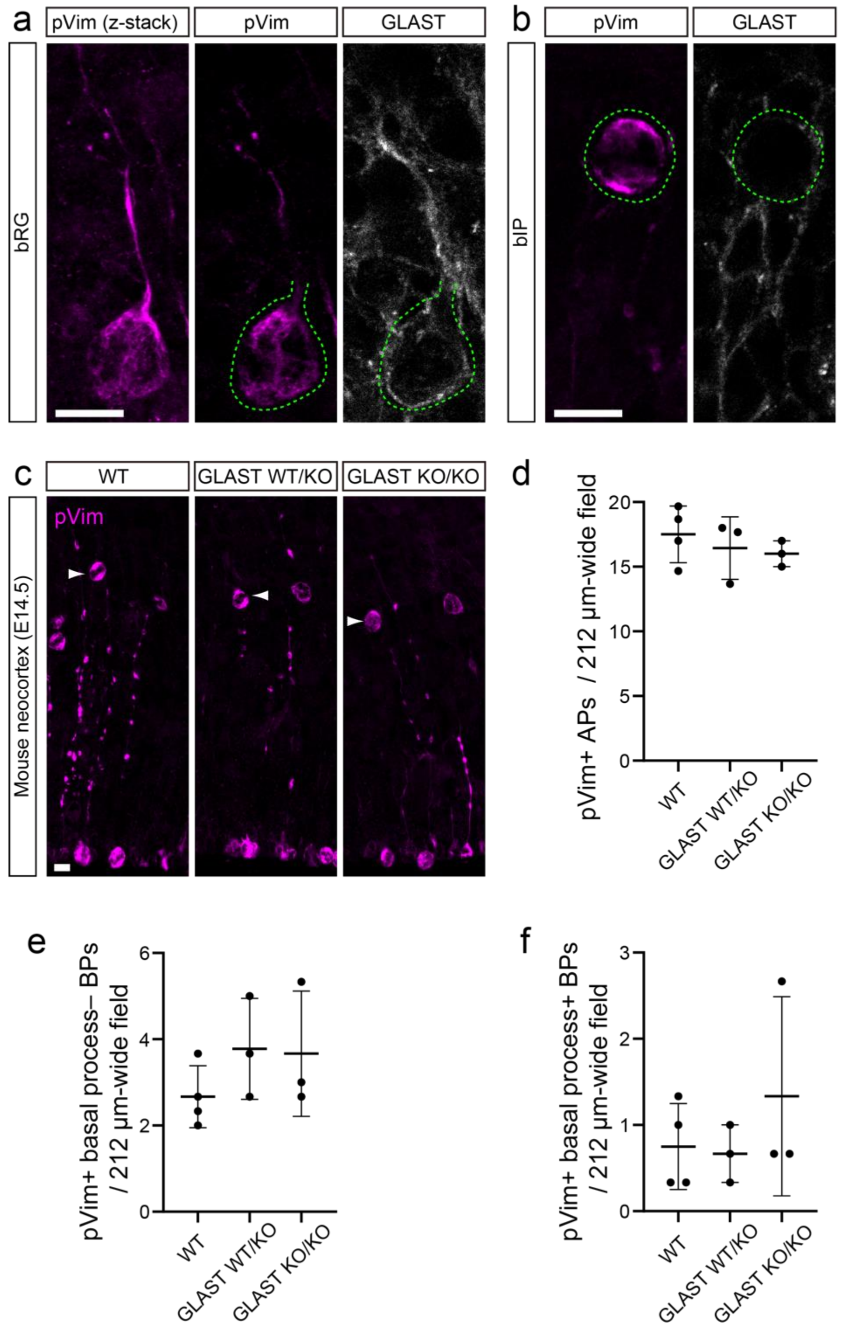

Supplemental figure 6. GLAST knockout does not change mitotic AP, bIP and bRG abundance.

a, **b**, GLAST expression in a mitotic bRG (**a**) and bIP (**b**). Immunofluorescence of E14.5 wildtype mouse neocortex for phosphovimentin (pVim, magenta) and GLAST (white). The dashed lines and circles indicate the outline of the pVim+ somata.

c, Immunofluorescence of E14.5 wildtype (WT), heterozygous GLAST knockout (GLAST WT/KO) and homozygous GLAST knockout (GLAST KO/KO) mouse neocortex for phosphovimentin (pVim, magenta). Arrowheads indicate mitotic BPs.

d-f, Quantification of pVim+ APs (**d**), pVim+ bIPs (**e**, basal process– BPs) and pVim+ bRG (**f**, basal process+ BPs), using immunostained cryosections obtained as in (**c**).

Error bars, SD; Scale bars: 10 µm in **a-c**.

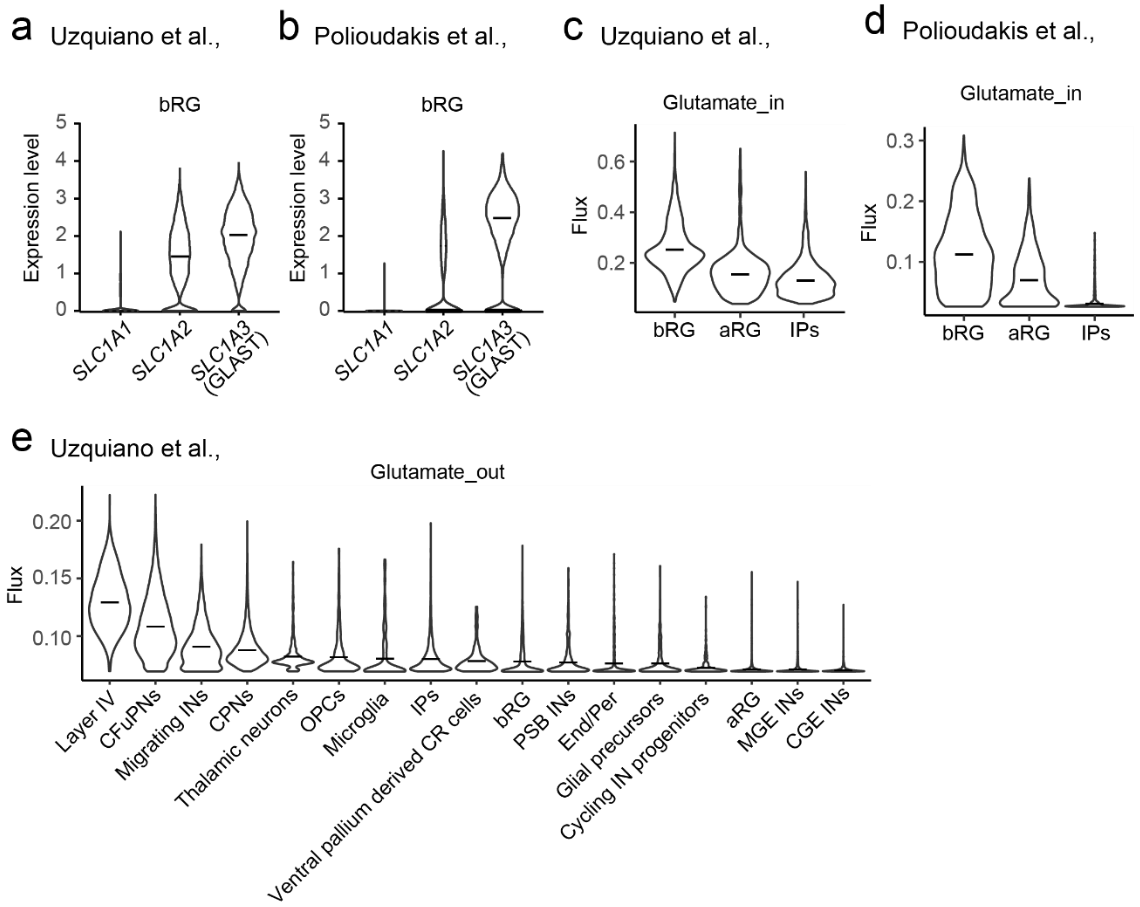

Supplemental figure 7. Glutamate flux estimation of NPCs and non-NPC–type cells in fetal human neocortex.

a-**d**, Glutamate transporter gene expression in bRG (**a**, **b**) and glutamate influx (Glutamate_in) score of bRG, aRG and IPs (intermediate progenitors) (**c**, **d**), which was calculated by single-cell flux estimation analysis (scFEA) based on the expression of *SLC1A1*, *SLC1A2*, *SLC1A3* (GLAST), *SLC1A5*, *SLC1A6* and *SLC1A7*. Analyses were done using single-nuclei/cell RNA sequencing data of fetal human neocortex at GW16-20[34] (**a**, **c**) and GW17-18[33] (**b**, **d**).

e, Glutamate efflux (Glutamate_out) score of the indicated cell types was calculated by scFEA based on the expression of *SLC17A6*, *SLC17A8* and *SLC17A7* obtained from single-nuclei/cell RNA sequencing data of fetal human neocortex at GW16-20[34].

Excitatory neurons: Layer IV (layer IV neurons), CFuPNs (corticofugal projection neurons), CPNs (callosal projection neurons), Thalamic neurons; NPCs: aRG (apical radial glia), bRG (basal radial glia), IPs (intermediate progenitors); interneurons (INs) and progenitors: Migrating INs, PSB (pallial-subpallial boundary) INs, cycling IN progenitors, MGE (medial ganglionic eminence) INs, CGE (caudal ganglionic eminence) INs; glial progenitors: OPCs (oligodendrocyte progenitor cells), Glial precursors; other cell types: Microglia, Ventral pallium derived CR (Cajal Retzius) cells, End/Per (endothelial/pericytes). Bars, mean.

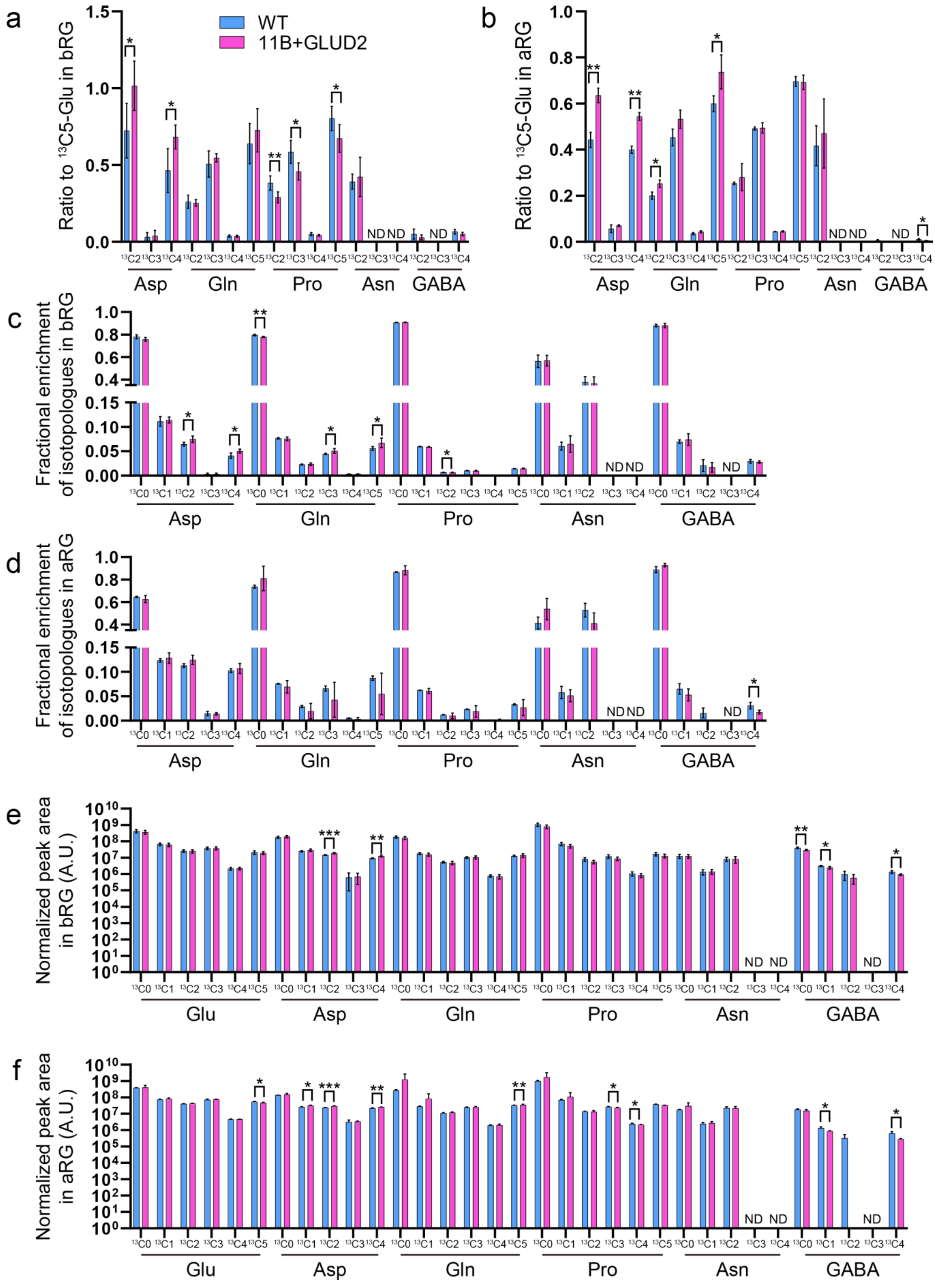

a
b
c
d
e
f
WT
11B+GLUD2
Ratio to ^{13}C5-Glu in bRG
Ratio to ^{13}C5-Glu in aRG
Fractional enrichment of isotopologues in bRG
Fractional enrichment of isotopologues in aRG
Normalized peak area in bRG (A.U.)
Normalized peak area in aRG (A.U.)
Glu
Asp
Gln
Pro
Asn
GABA
ND

Supplemental figure 8. Metabolic flux analysis of ^{13}C5-glutamate in bRG and aRG.

a, **b**, Peak areas of the indicated ^{13}C-labeled metabolites (Asp, aspartate; Gln, glutamine; Pro, proline; Asn, asparagine; GABA, γ-aminobutyric acid) are expressed as a ratio to the peak area of ^{13}C5-glutamate (^{13}C5-Glu) in bRG (**a**) and aRG (**b**) of WT (blue bars) and *ARHGAP11B+GLUD2* double-transgenic (magenta bars) mouse neocortex at E14.5.

c, **d**, Fraction of the indicated ^{13}C-labeled metabolites (Asp, aspartate; Gln, glutamine; Pro, proline; Asn, asparagine) in the total isotopologues of the indicated metabolites in bRG (**c**) and aRG (**d**) of WT (blue bars) and *ARHGAP11B+GLUD2* double-transgenic (magenta bars) mouse neocortex at E14.5.

e, **f**, Normalized peak areas (arbitrary units, A.U.) of the indicated ^{13}C-labeled metabolites (Glu, glutamate; Asp, aspartate; Gln, glutamine; Pro, proline; Asn, asparagine; GABA, γ-aminobutyric acid) in bRG (**e**) and aRG (**f**) of WT (blue bars) and *ARHGAP11B+GLUD2* double-transgenic (magenta bars) mouse neocortex at E14.5.

Error bars, SD; ND, not detected; $*p<0.05$; $**p<0.01$; $***p<0.001$; n = 5 (bRG) and n = 3 (aRG) biological replicates for the analysis.

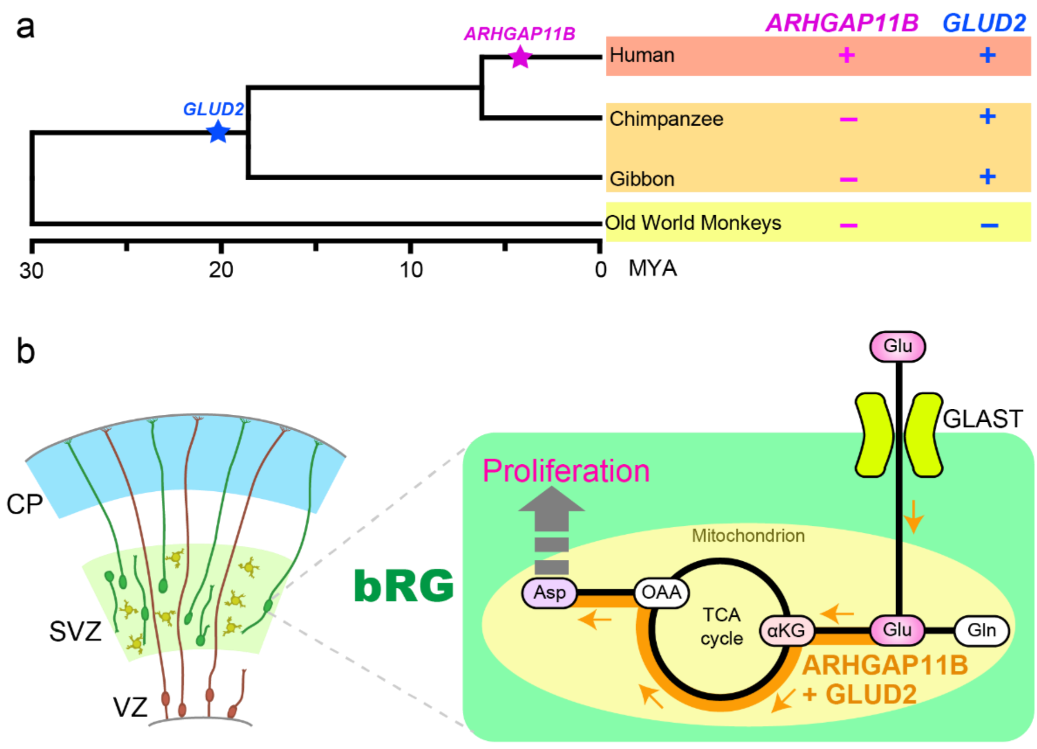

Supplemental figure 9. Emergence of *ARHGAP11B* and *GLUD2* during the evolution toward *Homo sapiens* (**a**), and their synergistic effect on bRG proliferation (**b**).

a, Phylogenetic tree showing the emergence of *ARHGAP11B* (~5 million years ago (MYA)) and *GLUD2* (18~23 MYA). *GLUD2* as an ape-specific gene is found in the genomes of the gibbon and the great apes including the genus *Homo*. *ARHGAP11B* as a human-specific gene is only found in the genomes of *homo*.

b, Right: Both ARHGAP11B and GLUD2 proteins are localized in mitochondria where they exert a functional synergy to promote the proliferative capacity of bRG. In addition to the glutaminolysis (glutamine (Gln) to glutamate (Glu) to alpha-ketoglutarate (αKG)) promoted by ARHGAP11B itself, the functional synergy of ARHGAP11B and GLUD2 utilizes glutamate (Glu), imported by GLAST (glutamate aspartate transporter) from the microenvironment, to produce aspartate (Asp) through alpha-ketoglutarate (αKG) via a three-quarter TCA cycle (orange) in bRG.

Left, from bottom to top: VZ (ventricular zone) with aRG (apical radial glia, red cells); SVZ (subventricular zone, green) with bRG (basal radial glia, green cells); CP (cortical plate, blue).
