# Supplementary material for: Functional synergy of a human-specific and an ape-specific metabolic regulator in human neocortex development
Source: Nat Commun. 2024 Apr 24;15:3468. doi: 10.1038/s41467-024-47437-8 (PMC11043075; doi:10.1038/s41467-024-47437-8)
Supplement: Supplementary file 3 — Reporting Summary [file 41467_2024_47437_MOESM3_ESM.pdf]

nature portfolio

Corresponding author(s): Takashi Namba

Last updated by author(s): 3/25/2024

Reporting Summary

Nature Portfolio wishes to improve the reproducibility of the work that we publish. This form provides structure for consistency and transparency in reporting. For further information on Nature Portfolio policies, see our Editorial Policies and the Editorial Policy Checklist.

Statistics

For all statistical analyses, confirm that the following items are present in the figure legend, table legend, main text, or Methods section.

n/a	Confirmed	
☐	☒	The exact sample size (*n*) for each experimental group/condition, given as a discrete number and unit of measurement
☐	☒	A statement on whether measurements were taken from distinct samples or whether the same sample was measured repeatedly
☐	☒	The statistical test(s) used AND whether they are one- or two-sided<br>*Only common tests should be described solely by name; describe more complex techniques in the Methods section.*
☒	☐	A description of all covariates tested
☐	☒	A description of any assumptions or corrections, such as tests of normality and adjustment for multiple comparisons
☐	☒	A full description of the statistical parameters including central tendency (e.g. means) or other basic estimates (e.g. regression coefficient) AND variation (e.g. standard deviation) or associated estimates of uncertainty (e.g. confidence intervals)
☐	☒	For null hypothesis testing, the test statistic (e.g. *F*, *t*, *r*) with confidence intervals, effect sizes, degrees of freedom and *P* value noted<br>*Give P values as exact values whenever suitable.*
☒	☐	For Bayesian analysis, information on the choice of priors and Markov chain Monte Carlo settings
☒	☐	For hierarchical and complex designs, identification of the appropriate level for tests and full reporting of outcomes
☒	☐	Estimates of effect sizes (e.g. Cohen's *d*, Pearson's *r*), indicating how they were calculated

Our web collection on statistics for biologists contains articles on many of the points above.

Software and code

Policy information about availability of computer code

Data collection	Zeiss ZEN 3.3
Data analysis	FiJi 1.54, Excel (Microsoft, Redmond, WA), Statcel3 (OMS, Japan), MYSTAT (Systat Software, CA) and GraphPad Prism 9 (GraphPad Software), the CoDEx viewer (http://solo.bmap.ucla.edu/shiny/webapp/), scFEA https://github.com/changwn/scFEA)

For manuscripts utilizing custom algorithms or software that are central to the research but not yet described in published literature, software must be made available to editors and reviewers. We strongly encourage code deposition in a community repository (e.g. GitHub). See the Nature Portfolio guidelines for submitting code & software for further information.

Data

Policy information about availability of data

All manuscripts must include a data availability statement. This statement should provide the following information, where applicable:

- Accession codes, unique identifiers, or web links for publicly available datasets
- A description of any restrictions on data availability
- For clinical datasets or third party data, please ensure that the statement adheres to our policy

All materials and data underlying the study are available from the corresponding authors with a completed Materials/Data Transfer Agreement.

Research involving human participants, their data, or biological material

Policy information about studies with human participants or human data. See also policy information about sex, gender (identity/presentation), and sexual orientation and race, ethnicity and racism.

Reporting on sex and gender	Gender is irrelevant for this study since we only used fetal tissues. Regarding the sex of samples, we did not assess the sex of mouse embryos used for experiments as we believe there is no sex difference in bRG abundance at this early stage during development when we performed our analysis. The sex of human fetal cortex could not be assessed because the informed consent form used in Finland did not indicate that the tissue would be used for chromosomal analyses. In this context, we would like to emphasize that mouse embryos and fetal human neocortical tissues were collected randomly. So any sex difference pertaining to our data would be included in the standard variation of our data.
Reporting on race, ethnicity, or other socially relevant groupings	No information about race, ethnicity, or other socially relevant grouping is allowed to be collected by the researcher.
Population characteristics	NA
Recruitment	The adult pregnant women seeking induced abortion at HUS will be approached by the clinical staff for recruitment as described below. The women will be provided both written and verbal essential information about the study at the time they visit the gynecological out-patients clinic or the fetal diagnostics unit. Abortion care will be provided at the hospital ward of the Department of Obstetrics and Gynecology according to Finnish legislation and National guideline in induced abortion at HUS as part of clinical routine. If the abortion is performed on social grounds, we will collect the results of the pre-abortion examinations, including fetal ultrasonography to verify that the fetus has no abnormalities.
Ethics oversight	This study is approved by the ethics committee of the Hospital district of Helsinki and Uusimaa (HUS/1170/2021)

Note that full information on the approval of the study protocol must also be provided in the manuscript.

Field-specific reporting

Please select the one below that is the best fit for your research. If you are not sure, read the appropriate sections before making your selection.

☒ Life sciences ☐ Behavioural & social sciences ☐ Ecological, evolutionary & environmental sciences

For a reference copy of the document with all sections, see nature.com/documents/nr-reporting-summary-flat.pdf

Life sciences study design

All studies must disclose on these points even when the disclosure is negative.

Sample size	No predetermination of sample sizes was carried out because our research is an exploratory study. The sample size has been decided based on the previous studies (e.g. Namba et al., Neuron 2020). All sample size are visible on the figures or figure legends.
Data exclusions	No data were excluded from the quantifications. If the tissue samples appeared to be severely damaged or dead, mouse or human tissue samples were excluded from further analyses.
Replication	All experiments were repeated at least three times, and all attempts were successful. All data from the replication were included in the analysis.
Randomization	Mouse embryos and human fetal tissues were randomly selected for all experiments and randomly allocated into the experimental groups.
Blinding	Sample collection and quantification was done blindly for the following image analysis: Figure 1, Figure 2c, e, g, h, Figure 3h, i, Figure 4, Supplemental fig. 2, Supplemental fig. 3, Supplemental fig. 4, Supplemental fig. 6. Supplemental fig. 1c and were done blindly. Metabolomics was done blindly by the core facility. Re-analyses of single cell and bulk transcriptome data were not done blindly since we need to know cell types to run the analyses. Supplemental fig. 1l was not done blindly since we needed to know that the cells are in the OSVZ.

Reporting for specific materials, systems and methods

We require information from authors about some types of materials, experimental systems and methods used in many studies. Here, indicate whether each material, system or method listed is relevant to your study. If you are not sure if a list item applies to your research, read the appropriate section before selecting a response.

Materials & experimental systems

n/a	Involved in the study
☐	☒ Antibodies
☐	☒ Eukaryotic cell lines
☒	☐ Palaeontology and archaeology
☐	☒ Animals and other organisms
☒	☐ Clinical data
☒	☐ Dual use research of concern
☒	☐ Plants

Methods

n/a	Involved in the study
☒	☐ ChIP-seq
☐	☒ Flow cytometry
☒	☐ MRI-based neuroimaging

Antibodies

Antibodies used	Antibodies used in this study were as follows; anti-ARHGAP11B (mouse IgG1, 3758-A37-5, MPI-CBG)21, anti-γ-tubulin (mouse IgG, T6557, Sigma), anti-GFP (goat IgG, MPI-CBG), anti-GFP (chicken IgY, GFP-1020, Aves), anti-GLAST (guinea pig IgG, GLAST-GP-Af1000, Frontier institute, Japan), anti-GLUD1/2 (rabbit IgG, ab166618, abcam), anti-Pax6 (rabbit IgG, 901301, BioLegend), anti-PCNA (mouse IgG, CBL407, Millopore), anti-PH3 (rat, ab10543, Abcam), anti-pVim (mouse IgG, D076-3, MBL), anti-SOX2 (goat IgG, AF2018, R&D Systems), anti-Tbr2 (rabbit IgG, ab23345, abcam), anti-TOM20 (mouse IgG, ab56783, Abcam), anti-TOM20 (rabbit IgG, ab78547, Abcam), anti-chicken IgY-Alexa Fluor 488 (donkey, 703-545-155, Jackson ImmunoResearch), anti-goat IgG-Alexa Fluor 488 (donkey, A11055, ThermoFisher Scientific), anti-goat IgG-Alexa Fluor 647 (donkey, A21447, ThermoFisher Scientific), anti-goat IgG-Alexa Fluor 647 (donkey, 705-605-147, Jackson ImmunoResearch), anti-guinea pig IgG-Aexa Fluor 647 (donkey, 706–605-148, Jackson ImmunoResearch), anti-mouse IgG-Alexa Fluor 488 (donkey, A21202, ThermoFisher Scientific), anti-mouse IgG-Alexa Fluor 555 (donkey, A31570, ThermoFisher Scientific), anti-mouse IgG-Alexa Fluor 647 (donkey, A31571, ThermoFisher Scientific), anti-mouse IgG-Cy3 (donkey, 715-165-151, Jackson ImmunoResearch), anti-mouse IgG-HRP (donkey, 715-035-151, Jackson ImmunoResearch), anti-rabbit IgG-Alexa Fluor 488 (donkey, A21206, ThermoFisher Scientific), anti-rabbit IgG-Alexa Fluor 488 (donkey, 711-545-152, Jackson ImmunoResearch), anti-rabbit IgG-Alexa Fluor 647 (donkey, A31573, ThermoFisher Scientific), anti-rabbit IgG-HRP (donkey, 711-035-152, Jackson ImmunoResearch).
Validation	All commercial antibodies are tested by the company as well as previous publications. Anti-GLUD2 antibody was produced using human GLUD2-specific peptide (PTAEFQDSISGA)46 in rabbit by Innovagen AB, Sweden. The specificity of rabbit polyclonal anti-GLUD2 (#13924.13), which was affinity purified by Protein-G and the GLUD2-specific peptide, was tested by Innovagen with ELISA using the GLUD2-specific peptide and GLUD1-specific peptide (PTAEFQDRISGA) (Fig. S1g). The specificity was also tested by immunoblot of cell lysate in which GLUD1 or GLUD2 were overexpressed (see below) (Fig. S1e, f). The specificity of other antibodies were done by the manufactures and available on their web site or data sheet.

Eukaryotic cell lines

Policy information about cell lines and Sex and Gender in Research

Cell line source(s)	COS-7 cells from ATCC
Authentication	The cell line was authenticated at least once by ATCC, and by us based on their morphology.
Mycoplasma contamination	Tested negative.
Commonly misidentified lines (See ICLAC register)	No commonly misidentified lines were used in this study.

Animals and other research organisms

Policy information about studies involving animals; ARRIVE guidelines recommended for reporting animal research, and Sex and Gender in Research

Laboratory animals	ARHGAP11B-transgenic mice, GLUD2 BAC transgenic mice (GLUD2-521177), C57BL/6N mice, all at E14.5. C57BL/6N mice at E13.5.
Wild animals	No wild animals were used in this study.
Reporting on sex	Sex of specimens has not been checked. All fetuses and embryos were collected randomly, therefore we expect to have both sex in the study.
Field-collected samples	No field-collected samples were used in this study.
Ethics oversight	All procedures regarding the animal experiments were approved by the Governmental agencies (Landesdirektion Sachsen, Germany; Aluehallintovirasto, Finland) and overseen by the Institutional Animal Welfare Officer(s). The license numbers concerning the experiments with mice are: Untersuchungen zur Neurogenese in Mäuseembryonen TVV2015/05 and ESAVI/15112/2021 (in utero electroporation) and 24–9168.24-9/2012-1 (tissue collection without prior in vivo experimentation).

Note that full information on the approval of the study protocol must also be provided in the manuscript.

Plants

Seed stocks	NA
Novel plant genotypes	NA
Authentication	NA

Flow Cytometry

Plots

Confirm that:

- [x] The axis labels state the marker and fluorochrome used (e.g. CD4-FITC).
- [x] The axis scales are clearly visible. Include numbers along axes only for bottom left plot of group (a 'group' is an analysis of identical markers).
- [x] All plots are contour plots with outliers or pseudocolor plots.
- [x] A numerical value for number of cells or percentage (with statistics) is provided.

Methodology

Sample preparation	Isolation of aRG and bRG was performed as previously described.11 Single-cell suspensions were prepared incubated embryonic mouse neocortical tissue using the MACS Neural Tissue Dissociation kit containing papain (Miltenyi Biotec) following the manufacturer's instruction. Cell-surface staining of prominin-1 (Prom-1) and GLAST was performed on the cell suspensions with rat 13A4 APC-conjugated antibody (1:50, eBioscience, Clone 13A4, #17-1331-81, RRID:AB_823120) and with anti-EAAT1/GLAST-1/SLC1A3 PE- conjugated antibody (1:10, Novus Biologicals, #NB100-1869PE).
Instrument	5-laser-BD FACSAria Fusion sorter (Becton Dickinson Biosciences)
Software	BD FACSDiva software
Cell population abundance	Sorting efficiency was generally more than 90%.
Gating strategy	First, a P1 gate was set on the SSC-A/FSC-A dot-plot, to identify live cells based on size and shape. Next, the P1 fraction was restricted by setting a P2 gate on the FSC- W/FSC-H dot-plot to select single cells. Out of the P2 population, single dot-plots were created for SSC-A/PE (linear/log2, yellow-green laser, 561 nm) and SSC-A/APC (linear/log2, red laser, 640 nm) to visualize the fluorescence intensities of Prom-1-APC and GLAST-PE, respectively. Voltage parameters were set based on an unstained control, and subsequently maintained for FACS. Next, the GLAST+/Prom-1+ gate was restrictively set and maintained through FACS to acquire aRG and the GLAST+/Prom-1– gate was restrictively set and maintained through FACS to acquire bRG, both of which were sorted at 4°C into 100 µl HBSS in Eppendorf tubes.

- [x] Tick this box to confirm that a figure exemplifying the gating strategy is provided in the Supplementary Information.
